# Prevalence of Sleep Apnea and Echocardiographic Correlates in a Community-Based Cohort of Cardio-Oncology Patients

**DOI:** 10.1016/j.jacadv.2025.102256

**Published:** 2025-10-28

**Authors:** Mini K. Das, John L. Huber, Jamie D. Kemp, Rebecca M. McFarland, Roberto Cardarelli, Sherill N. Cronin, Kenneth C. Anderson

**Affiliations:** a Baptist Health Medical Group Cardiology, Louisville, Kentucky, USA; b Baptist Health Medical Group Hematology & Oncology, Louisville, Kentucky, USA; c Baptist Health Louisville, Louisville, Kentucky, USA; d Lansing School of Nursing and Clinical Sciences, Bellarmine University, Louisville, Kentucky, USA; e Baptist Health Medical Group Sleep Medicine, Louisville, Kentucky, USA

**Keywords:** cardio-oncology, cancer therapy related cardiac dysfunction, left ventricular ejection fraction, left ventricular global strain, obstructive sleep apnea, STOP-Bang

## Abstract

**Background:**

Left ventricular ejection fraction (LVEF) and global longitudinal strain (GLS) predict cancer therapy-related cardiac dysfunction (CTRCD). Obstructive sleep apnea (OSA) contributes to LV dysfunction, abnormal GLS and heart failure, but has not been well-studied in cardio-oncology (CO) patients and is absent from CTRCD risk assessment tools.

**Objectives:**

The purpose of this paper was to evaluate the prevalence of OSA in a community-based CO population and the association to echocardiographic measures for CTRCD.

**Methods:**

Cross-sectional data including prevalence of conventional cardiovascular (CV) risk factors and sleep apnea status were collected in CO patients (n = 218). Pretreatment LVEF and GLS were obtained in these patients. Multivariable adjusted ORs (logistic regression) were calculated to assess the association between OSA status and pretreatment LVEF and GLS.

**Results:**

In CO patients, prevalence of OSA (38.5%, 84/218) was high and more frequent than many other CV risk factors. Baseline abnormal GLS, not an abnormal LVEF, was significantly different between the four subgroups: low OSA risk (prior negative sleep study or a low snoring, tiredness, observed apnea, and high blood pressure-body mass index, age, neck circumference, and gender score), treated OSA, untreated OSA, high OSA risk (a high snoring, tiredness, observed apnea, and high blood pressure-body mass index, age, neck circumference, and gender score) but a sleep study was not performed due to patient preference. In multivariable models, the presence of untreated OSA showed the strongest association with abnormal GLS (OR: 4.13; 95% CI: 1.32-12.93) when compared to other CV risk factors in CO patients.

**Conclusions:**

These observations suggest OSA be considered in risk algorithms for CTRCD. Larger studies are needed to corroborate these findings and assess the impact of OSA prevalence and treatment on outcomes in this high-risk population.

## Central Illustration

**Figure:**
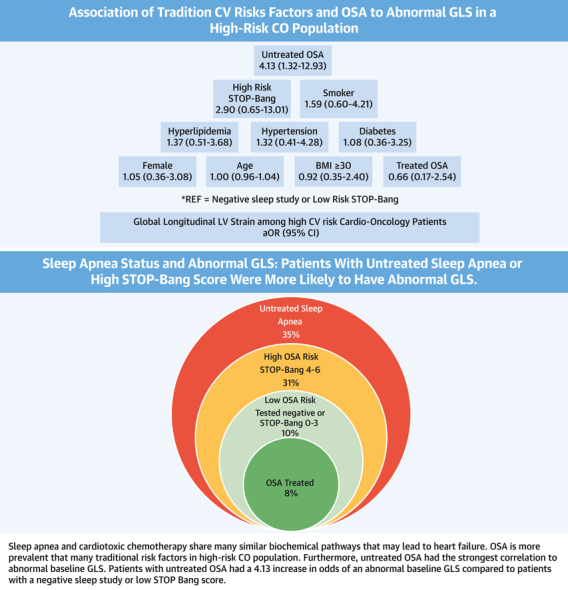

Obstructive sleep apnea (OSA) is characterized by repetitive episodes of partial or complete airway obstruction during sleep with associated apnea and hypopnea. OSA is an under-recognized risk factor for heart failure with increased morbidity, mortality, readmission rates, and increased health care costs. The reported prevalence of OSA varies greatly ranging from 9% to 37% in men and 4% to 50% in women in community cohorts.^1^, ^2^, ^3^ More recently, a large study of 20,151 participants from France using the Berlin Questionnaire demonstrated a total weighted prevalence of treated sleep apnea or high risk for sleep apnea of 21%.^4^ In patients with heart failure, either heart failure with reduced ejection fraction or heart failure with preserved ejection fraction, roughly 1 in 2 patients (52% and 48% respectively) carry a diagnosis of OSA where the treatment can improve survival.^1^^,^^3^ However, the prevalence of OSA has not been well studied in the growing population of oncology patients who have a high risk of cancer therapy-related cardiac dysfunction (CTRCD).

Left ventricular (LV) ejection fraction (LVEF) and global longitudinal strain (GLS) are 2 well-accepted echocardiographic measures to assess LV function that have been studied in OSA. GLS has been shown to be progressively abnormal with mild, moderate, and severe sleep apnea.^5^ This correlation has not been seen with LVEF.^5^ Early studies also suggest there are shared biochemical markers observed with untreated OSA and certain cancers that are targeted by specific cancer therapeutics.^1^^,^^6^, ^7^, ^8^, ^9^ This raises the concern that OSA may play a role in CTRCD.

The past 2 decades have witnessed a major expansion of effective cancer therapeutics that have increased cancer survivorship.^10^, ^11^, ^12^ With this, there is recognition of increased cardiovascular (CV) adverse outcomes, particularly CTRCD, and overt heart failure (HF) with cancer therapeutics. Shared risk factors for cancer and increased CV risk are also recognized.^13^, ^14^, ^15^ CTRCD is now well-established with multiple expert consensus papers and guidelines to address early identification and intervention of these shared CV risk factors to minimize the risk of CV events and avoid interruption of life saving cancer interventions.^13^, ^14^, ^15^ The purpose of our study was to determine the prevalence of OSA in a community-based cohort of cardio-oncology (CO) patients with increased CV risk and to examine the association of OSA, or a high likelihood of OSA, to current echocardiographic markers of CTRCD.

## Methods

From January 2022 to June 2023, we performed a retrospective chart review study of 218 patients who were consecutively evaluated in our CO program through referrals from medical oncologists, radiation oncologists, and breast surgeons before the initiation of cancer chemotherapy. The electronic medical record was used to determine the presence of risk factors previously accepted as shared clinical risk factors for CTRCD.^13^, ^14^, ^15^ These included hypertension, hyperlipidemia, diabetes, smoking, obesity, renal insufficiency, cancer diagnosis, prior stroke, prior myocardial infarction, prior HF, prior atrial fibrillation, gender, and age. The American College of Cardiology risk assessment format^13^^,^^16^ was used to stratify CV risk for cardiotoxicity in this CO group. Risk stratification using the European Society of Cardiology Guidelines^15^ was not applied as these guidelines were published after the initiation of this study.

Pretreatment echocardiograms were obtained in all CO patients and 200 (91.7%) had images that were adequate for assessment of LVEF and GLS. These are 2 validated parameters for early identification of LV dysfunction used to guide the therapy to minimize CV risk and avoid interruption of life saving cancer therapy.^16^^,^^18^ Normal LVEF was defined as ≥50% and abnormal LVEF <50%. Normal GLS was defined as ≤−18% and abnormal GLS ≥−17.9%.^17^ Echocardiograms were performed using the Phillips EPIC CVx echo machine, software level 9.0 with transducer X5-1.

In our CO practice, as part of our protocol we routinely administer a sleep questionnaire to all CO patients to assess OSA risk as these patients receive potential cardiotoxic interventions. The sleep questionnaire is used to determine if patients have had a prior evaluation for sleep apnea (treated or untreated) and if no prior evaluation had been obtained, a STOP-Bang (snoring, tiredness, observed apnea, and high blood pressure-body mass index, age, neck circumference, and gender) score was administered. The STOP-Bang score is an established and well-validated method of screening for sleep apnea.^6^^,^^19^, ^20^, ^21^ The STOP-Bang questionnaire consists of 8 questions. It includes four yes/no clinical options: STOP: snoring, tiredness, observed apnea, and high blood pressure and 4 demographic options: Bang: BMI, age, neck circumference, and gender. A STOP-Bang score of ≥3 has a high sensitivity (90% for moderate OSA and 100% for OSA). However, the specificity is low (47% for moderate OSA and 37% for severe OSA). We therefore used a STOP-Bang score ≥4 with lower sensitivity but greater specificity for moderate to severe OSA.^20^ A patient-reported history of sleep apnea with a prior diagnostic sleep study was used to define presence of diagnosed OSA. If patients were not previously tested, a STOP-Bang score was obtained. Four OSA risk subgroups were categorized. The first subgroup, low OSA risk, consisted of patients with prior negative sleep study or a low STOP-Bang score (STOP-Bang score ≤3). The second subgroup, treated OSA, consisted of patients who reported they were treating previously diagnosed sleep apnea. The third subgroup, untreated OSA, consisted of patients who reported they were not treating previously diagnosed OSA. The fourth subgroup consisted of patients with a high STOP-Bang score (STOP-Bang score ≥4), but the sleep study was not performed due to patient preference.

### Statistical Analysis

The prevalence of the traditional CV risk factors and OSA risk in this group of CO patients was assessed. The association between OSA status and traditional CV risk factors to abnormal LVEF and GLS was tested using logistic regression. Models were adjusted for age alone (model 1) and other CV risk factors previously listed in (model 2). A *P* value <0.05 was used for all analyses to establish statistical significance.

This study was approved by the Baptist Health Lexington Institutional Review Board.

## Findings

As expected in a referral population of CO patients, 95% were at increased ASCVD risk using the American College of Cardiology risk assessment format.^13^^,^^16^ The distribution of CV risk factors in the CO group is shown in Table 1. In this group the average age is 65.7 years and 78.9% (172/218) are female. The incidence of hypertension is 63.3% (138/218), hyperlipidemia is 52.8% (115/218), high risk of OSA (prior history of OSA or an elevated STOP-Bang score) is 46.3% (101/218), obesity is 43.6% (95/218), prior history of OSA is 38.5% (84/218), smoking is 32.6% (71/218), renal insufficiency is 31.7% (69/218), diabetes is 25.2% (55/218), atrial fibrillation is 9.2% (20/218), HF is 7.8% (17/218), and myocardial infarction is 2.8% (6/218). Approximately 1 in 3 CO patients had a diagnosis of OSA, and nearly 1 in 2 were at a high risk for OSA when considering the STOP-Bang score. The presence of diagnosed OSA or high risk of OSA was greater than many of the other CV risk factors.

**Table 1:** Characteristics of Cardio Oncology Patients

	Cardio-Oncology Patients (n = 218)	95% CI
Age, y, mean (SD)	65.7 (14.4)	50.41-80.99
Female, n (%)	172 (78.9)	72.88-84.12
Smoking, n (%)	71 (32.6)	26.39-39.23
Obesity (BMI ≥30 kg/m^2^), n (%)	95 (43.6)	36.89-50.54
Hypertension, n (%)	138 (63.3)	56.53-69.71
Hyperlipidemia, n (%)	115 (52.8)	45.90-59.53
Diabetes, n (%)	55 (25.2)	19.61-31.54
Renal insufficiency, n (%)	69 (31.7)	25.54-38.27
Atrial fibrillation	20 (9.2)	5.69-13.81
Myocardial infarction, n (%)	6 (2.8)	1.02-5.89
Heart failure, n (%)	17 (7.8)	4.61-12.19
Previous OSA diagnosis, n (%)	84 (38.5)	32.04-45.34
Treated	55	
Untreated	29	
OSA risk groups		
Low: no prior OSA or STOP-Bang 0-3	117 (53.7)	46.81-60.43
High: prior OSA or STOP-Bang 4-6	101 (46.3)	39.57-53.19

Prevalence of risk factors in cardio-oncology.

BMI = body mass index; OSA = obstructive sleep apnea; STOP-Bang = snoring, tiredness, observed apnea, and high blood pressure-body mass index, age, neck circumference, and gender.

**Figure 1:**
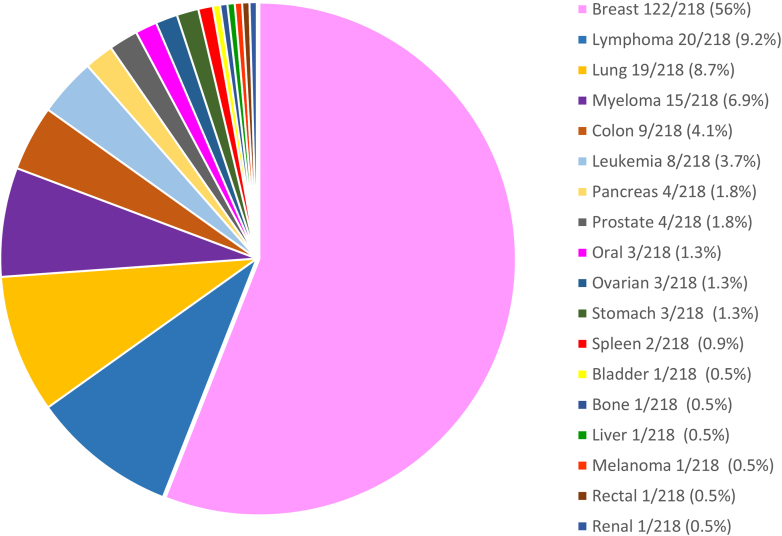
**Study Population Cancer Types** Prevalence of study population cancer types.

The prevalence of types of cancer treated in this population is shown in Figure 1. These included a prevalence of breast cancer is 56% (112/218), lymphoma is 9.2% (20/218), lung cancer is 8.7% (19/218), myeloma is 6.9% (15/218), colon cancer is 4.1% (9/218), leukemia is 3.7% (8/218), pancreatic cancer is 1.8% (4/218),prostate cancer is 1.8% (4/218), oral cancer is 1.3% (3/218), ovarian cancer is 1.3% (3/218), and stomach cancer is 1.3% (3/218). The remaining 3.7% (8/218) were comprised of splenic, bladder, bone, liver, rectal, renal carcinoma, and melanoma.

**Figure 2:**
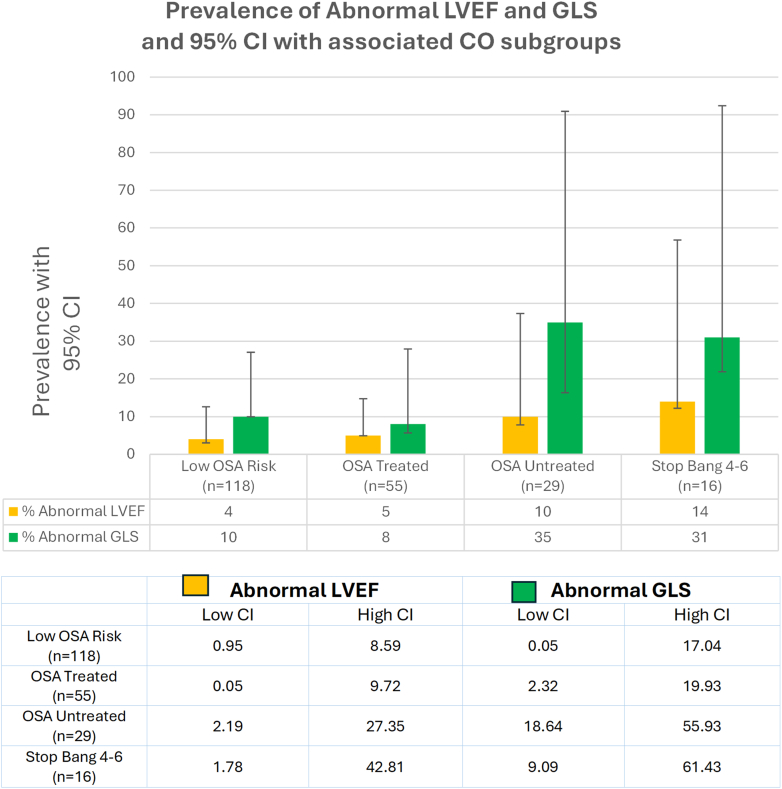
**Prevalence of Abnormal Left Ventricular Ejection Fraction and Global Longitudinal Strain in Cardio-Oncology Subgroups With Associated 95% CI Plot** Comparison of LVEF and GLS in cardio-oncology patients separated by sleep apnea status: patients at low OSA risk (negative sleep study or a low STOP-Bang score), patients with treated OSA, patients with untreated OSA and patients with a high STOP-Bang score who are at high risk for sleep apnea but not tested. The 95% CIs are superimposed on the graph. There is a wider range noted for the untreated and high Stop-Bang that was a smaller sample size. GLS = global longitudinal strain; LVEF = left ventricular ejection fraction; OSA = obstructive sleep apnea; CO = cardio-oncology.

Among the 218 patients in the CO group, there was adequate visualization of LVEF and GLS in 200 patients (91.7% with pretreatment values). Of these 200 patients, 10 patients (5%) had an abnormal LVEF <50% and 29 patients (14.5%) had an abnormal GLS ≥−17.9%. We observed that there were differences in LVEF and GLS among patients with untreated sleep apnea or a high STOP-Bang score compared to patients with treated OSA or with a low STOP-Bang score (Figure 2). ORs were calculated separately for abnormal LVEF (Table 2) and GLS (Table 3) by sleep apnea subgroups and adjusted for age (model 1) and for traditional CV risk factors (model 2). LVEF was not associated with untreated or treated sleep apnea, or with a high STOP-Bang score compared to those with negative sleep study/low STOP-Bang score (Table 2). GLS was significantly associated with untreated sleep apnea compared to patients with a negative sleep study/low STOP-Bang score (Table 3). Abnormal GLS was associated with a high STOP-Bang score or untreated OSA in either model when compared to those with a negative sleep study/low STOP-Bang score or treated OSA. In the age-adjusted model (Table 3) (model 1), patients with untreated sleep apnea had a 4.65 increase in odds of an abnormal GLS (OR: 4.65; 95% CI: 1.69, 12.80) compared to those with a negative sleep study/low STOP-Bang score. When adjusted for other CV traditional risk factors (Table 3) (Model 2), this significant association remained (OR: 4.13; 95% CI: 1.32, 12.93).

**Table 2:** Abnormal Left Ventricular Ejection Fraction Among Cardio-Oncology Patients

Characteristics	Model 1	Model 2
OSA risk status subgroups		
Negative sleep study or low-risk STOP-Bang (n = 110)	REF	REF
High-risk STOP-Bang (n = 14)	5.71 (0.86-37.79)	3.51 (0.46-26.85)
Treated OSA (n = 48)	0.72 (0.07-7.15)	0.66 (0.06-7.94)
Untreated OSA (n = 28)	3.77 (0.70-20.34)	2.84 (0.42-18.98)
Age	1.02 (0.96-1.08)	1.04 (0.97-1.11)
Female	--	0.37 (0.07-1.86)
History of hypertension	--	0.34 (0.05-2.22)
History of MI	--	--
Smoker	--	0.90 (0.17-4.66)
BMI ≥30, kg/m^2^	--	1.33 (0.29-6.14)
History of heart failure	--	--
History of diabetes	--	2.21 (0.32-15.43)
History of hyperlipidemia	--	0.70 (0.14-3.58)

Values are aOR (95% CI). Comparison of baseline LVEF vs patient risk factors incorporating sleep apnea status corrected for age in model 1 and corrected for other traditional risk factors in model 2.

aOR = adjusted OR; MI = myocardial infarction; other abbreviations as in Table 1.

**Table 3:** Abnormal Global Longitudinal Strain Among Cardio-Oncology Patients

Characteristics	Model 1	Model 2
OSA risk status subgroups		
Negative sleep study or ow-risk STOP-Bang (n = 110)	REF	REF
High-risk STOP-Bang (n = 14)	3.50 (0.94-13.08)	2.90 (0.65-13.01)
Treated OSA (n = 48)	0.79 (0.24-2.64)	0.66 (0.17-2.54)
Untreated OSA (n = 28)	4.65 (1.69-12.80)	4.13 (1.32-12.93)
Age	1.01 (0.98-1.04)	1.00 (0.96-1.04)
Female	--	1.05 (0.36-3.08)
History of hypertension	--	1.32 (0.41-4.28)
History of MI	--	--
Smoker	--	1.59 (0.60-4.21)
BMI ≥30, kg/m^2^	--	0.92 (0.35-2.40)
History of heart failure	--	--
History of diabetes	--	1.08 (0.36-3.25)
History of hyperlipidemia	--	1.37 (0.51-3.68)

Values are aOR (95% CI). Comparison of baseline GLS vs patient risk factors incorporating sleep apnea status corrected for age in model 1 and corrected for other traditional risk factors in model 2. The age-adjusted model illustrated those patients with untreated sleep apnea had a 4.65 increase in odds of an abnormal GLS compared to those with a negative sleep study/low STOP-Bang score. When adjusted for other CV traditional risk factors this significant association remained.

Abbreviation as in Tables 1 and 2.

## Discussion

This study (Central Illustration) serves as one of the first community-based studies evaluating the prevalence of sleep apnea in an increased ASCVD risk CO population and its association with CV functional markers. This study’s CO population had a higher prevalence of diagnosed OSA (38.5%, 84/218) compared to previous general population estimates.^2^, ^3^, ^4^^,^ When considering those with a high STOP-BANG score, the prevalence increased further to 46.3%. The pretreatment echo findings in this CO population are consistent with echo findings in the general sleep apnea population previously reported by Tadic et al.^5^ with better correlation of GLS than LVEF with treated vs untreated OSA. Similarly in this study, we found CO patients with untreated sleep apnea were more likely to have an abnormal GLS than patients with treated sleep apnea in this population. The strength of this association was greater than for established CV risk factors that currently comprise risk algorithms for CTRCD. Our study identifies a prevalent risk factor in this population previously not recognized and furthermore our findings suggest a strong correlation to OSA status (treated vs untreated).

The high prevalence of OSA and associated HF is important to recognize as despite advances in drug regimens and mechanical interventions, HF remains a major health problem with high morbidity and mortality rates in the general population.^7^^,^^8^ The current rate of HF among U.S. adults is approximately 1.9% to 2.6%.^7^^,^^8^ This prevalence of overt HF which relies on cases coming to medical attention underestimates the true prevalence of HF, which is often subclinical.^7^^,^^8^ In parallel, with the growth of cancer therapeutics with increased survivorship is the emergence of cardiotoxicity from these interventions, particularly cardiomyopathy. Symptomatic HF or decline in EF has been noted to occur at a rate of 5%^10^, ^11^, ^12^, ^13^ and with inclusion of other markers (high sensitivity troponin T and N-Terminal natriuretic pro-peptide), in some subgroups up to 37.5%.^14^ The recognition and intervention with cardioprotective therapy may have the potential to lower this event rate and impact premature interruption or cessation of life-saving cancer therapy.^13^, ^14^, ^15^

With the advent of strain imaging, subclinical cardiomyopathy can be detected before the decline of LV function, and it is routinely used to detect early cardiotoxicity.^16^^,^^18^ LVEF and resting GLS are now central to assessment before, during, and after cancer therapy with EF <50% or GLS >−18% as markers of cardiotoxicity.^9^^,^^17^^,^^22^^,^^23^ In addition, change in GLS of >15%, even with a normal LVEF, is consistent with probable early subclinical cardiomyopathy.^15^ By using the standard cutoff of 50% LVEF to define LV dysfunction we may be underestimating mild subclinical cardiomyopathy with LVEF of 50% to 55% which may correlate more robustly with LVEF vs GLS in this population. Cardiac biomarkers may help to clarify this subgroup of patients further and should be considered in future studies assessing CV risk factors, in particular, sleep apnea. More recently other echo parameters such as global work index and global constructive work have been shown to correlate well to other prognostic parameters of HF and to severity of sleep apnea.^24^, ^25^, ^26^, ^27^, ^28^ GLS, global work index, and global constructive work have also been shown to be progressively abnormal with mild, moderate, or severe sleep apnea.^26^, ^27^, ^28^ Importantly, GLS and LV global work efficiency have also been shown to improve with continuous positive airway pressure therapy.^25^^,^^27^^,^^28^

Sleep apnea has clearly been shown to contribute to the progression of HF with cumulative biochemical changes with increased plasma catecholamine and sympathetic nerve activation (Central Illustration).^1^^,^^3^ This leads to increased oxidative stress, inflammation, monocyte invasion into the endothelium, and subsequent endothelial dysfunction. It is hypothesized that through preconditioning of the myocardium by endothelial dysfunction with impaired vasodilation, decreased availability of nitrous oxide, and increased vasoconstriction, there is an increase in endothelin-1, interleukin-6 (IL-6), interleukin-8 (IL-8), programmed cell death-ligand 1 (PD-L1), and tumor necrosis factor–alpha (TNF-α).^1^^,^^29^^,^^30^ A recent study by Pries et al. showed treatment of OSA with hypoglossal nerve stimulation improved the expression of adhesion molecule CD162 on monocytes with downregulation of the checkpoint molecule PD-L1 on three monocyte subsets.^31^ The PD-L1 molecule is also a target of immune checkpoint therapy which has revolutionized cancer therapy.^34^ This implies a potentially important biochemical link of OSA, LV dysfunction, cancer, and cancer therapy. This raises the question whether the presence of OSA may further potentiate cardiotoxicity from cancer therapeutic interventions. Simultaneously, treatment of sleep apnea (with unintentional weight loss or mechanical intervention) may attenuate this preconditioning as suggested by the lower rates of abnormal strain at rest in patients with treated sleep apnea as found in this study and other studies of the general population.^28^ Sleep apnea is a treatable and prevalent entity with many reversible CV endpoints with associated impact on echo parameters (GLS more so than LVEF) and where self-correction or exacerbation with weight loss or weight gain, respectively, often seen during cancer therapy may occur in this population. This raises the question of what contribution this highly prevalent risk factor may have to future outcome studies using strain and EF parameters to guide cardioprotective intervention in patients receiving cardiotoxic chemotherapy.

**Central Illustration:**
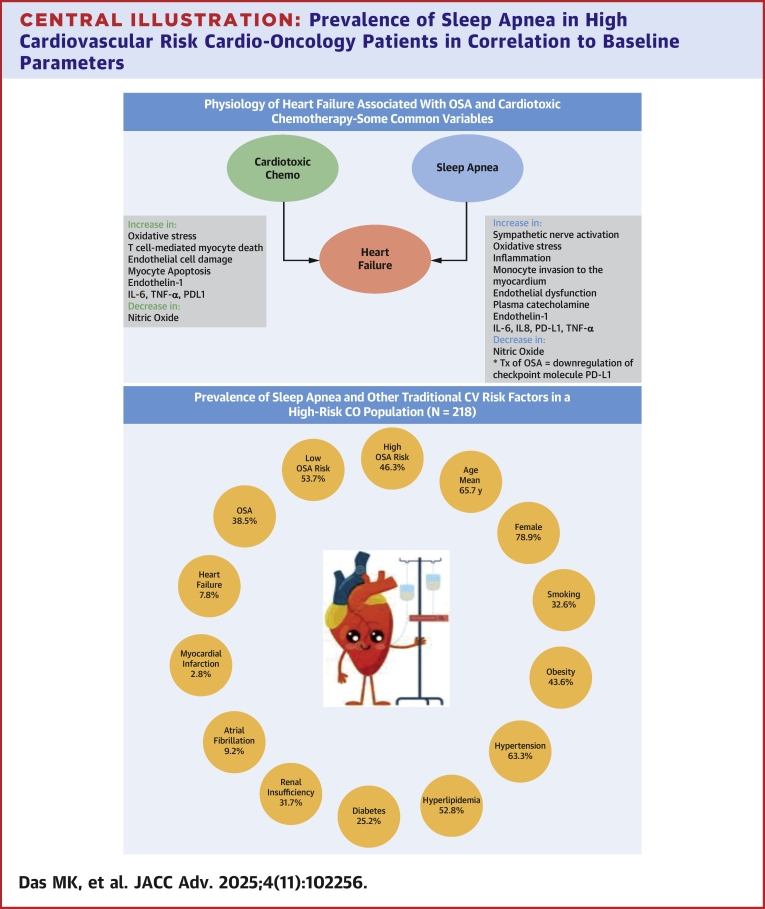

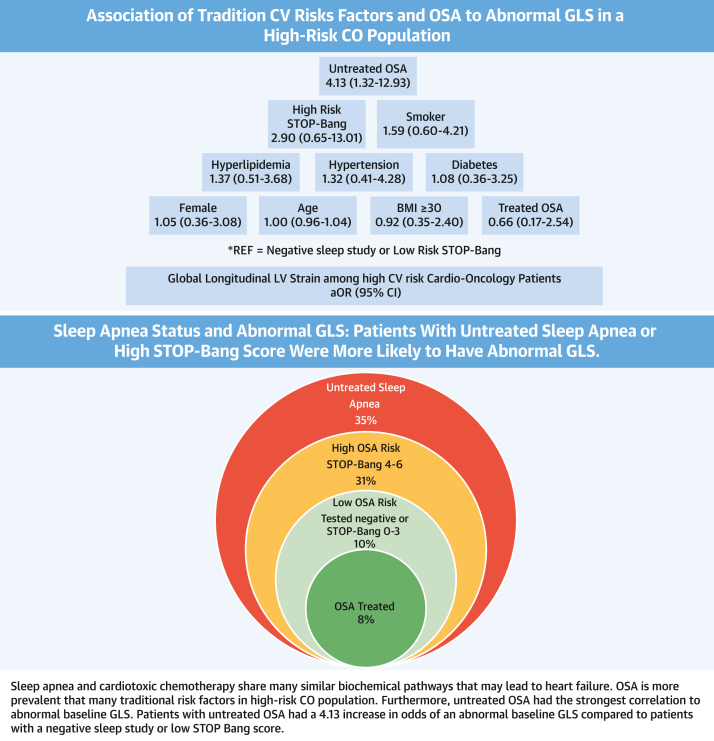
**Prevalence of Sleep Apnea in High Cardiovascular Risk Cardio-Oncology Patients in Correlation to Baseline Parameters** Sleep apnea and cardiotoxic chemotherapy share many similar biochemical pathways that may lead to heart failure. OSA is more prevalent that many traditional risk factors in high-risk CO population. Furthermore, untreated OSA had the strongest correlation to abnormal baseline GLS. Patients with untreated OSA had a 4.13 increase in odds of an abnormal baseline GLS compared to patients with a negative sleep study or low STOP Bang score. Heart cartoon image by Olga Kurbatova.

Currently, none of the consensus statements or guidelines address sleep apnea despite the strong association of sleep apnea to HF and other adverse CV events. We believe this is because of paucity of data in this vulnerable population. The current study provides new insight on the potential large impact of OSA on CV health in the high-risk cardio-oncologic population.

Larger prospective studies using traditional CV risk factors, sleep apnea classification (with treated, untreated sleep apnea or high STOP-Bang score), echo parameters (including LVEF, GLS and LV myocardial work), and biomarkers are warranted for this population. Whether the impact of LV strain or various parameters of LV myocardial work will be more discriminatory than LVEF in CO population on therapeutic outcomes when taking sleep apnea and its treatment into account, is yet to be determined.

### Study limitations

This community-based study’s limitations include a small sample size, gender bias, heterogeneity in cancer types, and lack of randomized comparison groups. This is a retrospective observational study of patients in a clinical community practice and additional datapoints needed for a prospective outcomes study were not completely obtained in all patients. However, this study indicates that sleep apnea remains highly prevalent in this population of patients with increased CV risk referred to CO programs and who are subjected to life-saving cancer therapeutic interventions with potentially adverse CV outcomes. This study also suggests that there is a potentially strong association of sleep apnea to current day tools used to assess baseline cardiac risk (GLS and LVEF).

As with any study using patient reported data, there is some inherent potential for subjective errors in patient reporting. This study, however, reflects a pragmatic assessment and treatment of sleep apnea using a well-established screening tool, STOP-Bang score. Because of these limitations and paucity of currently available data in this area, further evaluation with larger prospective trials is warranted in patients who have shared risk factors for adverse CV events and may benefit significantly from recognition and treatment of sleep apnea. Sleep apnea is fortunately a treatable condition that decreases CV events and improves various echo parameters.^1^^,^^28^^,^^29^^,^^32^ We acknowledge our limitations and await prospective studies to address the impact of treating sleep apnea on CV outcomes and possible variations that may surface in various drug regimens in this population of patients with an increased CV risk.

Perspectives **COMPETENCY IN MEDICAL KNOWLEDGE:** Sleep apnea is a highly prevalent finding in HF within the general population and treatment can improve HF with positive impact on echo correlates of LVEF and GLS. CTRCD is an entity that can impede cancer therapy and can impact long-term CV outcomes and is also associated with echo correlates LVEF and GLS. However, the current risk assessments algorithms to determine CTRCD do not take into account untreated OSA. We found in this study that the prevalence of sleep apnea is greater than reported in prior general population studies (38.5% vs21%). Furthermore, in this population this risk factor is more prevalent than many of the traditional risk factors currently used for risk stratification for CTRCD. This study also showed that untreated sleep apnea had the strongest association to abnormal baseline GLS when compared to other traditional CV risk factors in this community-based CO population. Implications of this study suggest that this prevalent and modifiable risk factor should be considered in algorithms for the baseline CV risk assessment for CTRCD.

**TRANSITIONAL OUTLOOK:** We found the sleep apnea questionnaire, including the STOP-Bang score, to be an easy tool to apply at baseline visits regardless of gender, age, ethnicity, or social status. However, access remains a problem with many communities that lack a CO program. If sleep apnea is incorporated as a risk factor in future algorithms for CTRCD, sleep questionnaires can be used by general cardiologists and oncologists. Another challenge to care is the timeliness of addressing untreated sleep apnea early in cancer therapeutics. Hopefully, with the data suggested in this study, access and utilization of sleep medicine should be considered more frequently and earlier in care for these higher risk individuals. Another limitation is the lack of understanding of sleep related hypoxia and related vasoconstriction, increased oxidative stress and endothelial dysfunction at the molecular level for various chemotherapeutic agents. Additional research is needed to incorporate this risk factor in risk assessment and whether treatment of sleep apnea will impact outcomes for CO patients that are at an increased risk for CTRCD. This additional research will need to include adequacy of sleep apnea therapy and be large enough to hopefully consider biochemical differences in varied chemotherapeutic protocols.

## Funding support and author disclosures

Funds were awarded through Baptist Health Greater Louisville Foundation (award ID: LOU26282). The authors have reported that they have no relationships relevant to the contents of this paper to disclose.
